# Supplementary material for: The Risk of Venous Thromboembolism in Korean Patients with Breast Cancer: A Single-Center Experience
Source: Cancers (Basel). 2023 Jun 9;15(12):3124. doi: 10.3390/cancers15123124 (PMC10296218; doi:10.3390/cancers15123124)
Supplement: Supplementary file 1 [file cancers-15-03124-s001.zip › cancers-2393851-supplementary.pdf]

Supplementary Figure S1. Comparison between patients with asymptomatic VTE and symptomatic VTE. There were no significant differences in disease-free survival (*p*=0.558) and overall survival (*p*=0.328).

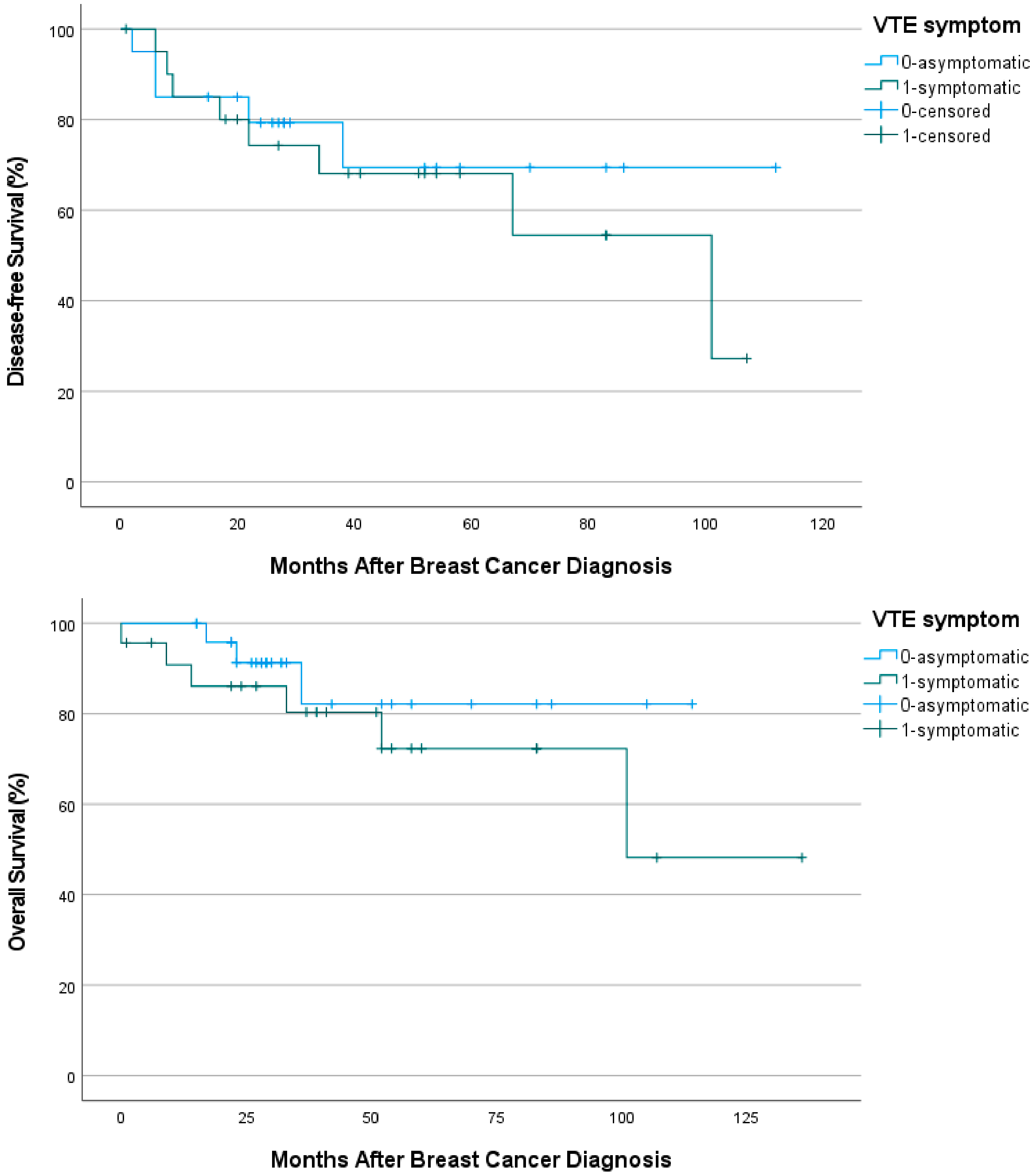

Supplementary Table S1. The association between comorbidities and VTE

	Patients without VTE (n=2198)	Patients with VTE (n=48)	*p*
Hypertension			<0.001
Absent	1675 (76.2%)	25 (52.1%)	
Present	523 (23.8%)	23 (47.9%)	
Diabetes mellitus			0.020
Absent	1995 (90.8%)	38 (79.2%)	
Present	203 (9.2%)	10 (20.8%)	
Malignancy other than breast cancer			0.289
Absent	2094 (95.3%)	44 (91.7%)	
Present	104 (4.7%)	4 (8.3%)	
Psychiatric disorder			0.155
Absent	2137 (97.2%)	45 (93.8%)	
Present	61 (2.8%)	3 (6.3%)	
Asthma or COPD			0.141
Absent	2169 (98.7%)	46 (95.8%)	
Present	29 (1.3%)	2 (4.2%)	
History of pulmonary tuberculosis			1.000
Absent	2156 (98.1%)	48 (100%)	
Present	42 (1.9%)	0	
History of stroke			0.490
Absent	2168 (98.6%)	47 (97.9%)	
Present	30 (1.4%)	1 (2.1%)	
Coronary artery disease			0.126
Absent	2171 (98.8%)	46 (95.8%)	
Present	27 (1.2%)	2 (4.2%)	
Chronic kidney disease			0.022
Absent	2189 (99.6%)	46 (95.8%)	
Present	9 (0.4%)	2 (4.2%)	
Parkinson's disease			1.000
Absent	2189 (99.6%)	48 (100%)	
Present	9 (0.4%)	0	
Liver cirrhosis			1.000
Absent	2192 (99.7%)	48 (100%)	
Present	6 (0.3%)	0	
Atrial fibrillation			1.000
Absent	2190 (99.6%)	48 (100%)	
Present	8 (0.4%)	0	
Heart failure			1.000
Absent	2193 (99.8%)	48 (100%)	
Present	5 (0.2%)	0	

COPD, chronic obstructive pulmonary disease

Supplementary Table S2-1. Comparison between asymptomatic and symptomatic VTE

	Asymptomatic VTE (n=25)	Symptomatic VTE (n=23)	*p*
Age, years			0.677
≤44	1 (4.0%)	2 (8.7%)	
45–59	10 (40.0%)	7 (30.4%)	
≥60	14 (56.0%)	14 (60.9%)	
Male sex	0	2 (8.7%)	0.001*
Comorbidity			0.616
Absent	20 (80.0%)	17 (73.9%)	
Present	5 (20.0%)	6 (26.1%)	
Operation			0.293
BCS	8 (34.8%)	8 (34.8%)	
TM without reconstruction	17 (68.0%)	13 (56.5%)	
TM with reconstruction	0	2 (8.7%)	
Bilaterality	2 (8.0%)	1 (4.3%)	1.000*
Stage			0.788
I	6 (24.0%)	5 (21.7%)	
II	11 (44.0%)	12 (52.2%)	
III	5 (20.0%)	5 (21.7%)	
IV	5 (20.0%)	5 (21.7%)	
Hormonal receptor			0.222*
Negative	6 (24.0%)	10 (43.5%)	
Positive	19 (76.0%)	13 (56.5%)	
HER2 status			0.332
Negative	14 (56.0%)	16 (69.6%)	
Positive	11 (44.0%)	7 (30.4%)	
Chemotherapy			0.407*
Not performed	2 (8.0%)	4 (17.4%)	
Performed	23 (92.0%)	19 (82.6%)	
Endocrine treatment			0.394
Not done	8 (32.0%)	11 (47.8%)	
Tamoxifen±OFS	6 (24.0%)	6 (26.1%)	
Aromatase inhibitor	11 (44.0%)	6 (26.1%)	

VTE, venous thromboembolism; BCS, breast conserving surgery; TM, total mastectomy; HER2, human epidermal growth factor receptor 2; OFS, ovarian function suppression
*Fisher's exact test

Supplementary Table S2-2. Factors associated with symptomatic VTE occurrence

	Incidence	HR (95% CI)	*p*
Age group			
≤44	0.4%	1	
45–59	0.6%	1.493 (0.310–7.185)	0.617
≥60	2.5%	6.737 (1.530–29.658)	0.012
Sex			
Female	0.9%	1	
Male	33.3%	40.506 (9.488–172.918)	<0.001
Operation			
BCS	0.8%	1	
Simple mastectomy	1.4%	1.866 (0.773–4.501)	0.165
Mastectomy with reconstruction	14.3%	23.570 (5.286–105.108)	<0.001
Stage			
0	0	0	0.975
I	0.6%	1	
II	1.6%	2.770 (0.976–7.863)	0.056
III	2.2%	3.856 (1.116–13.322)	0.033
IV	2.1%	3.771 (0.440–32.294)	0.226
Hormonal receptor			
Negative	1.9%	1	
Positive	0.8%	0.407 (0.179–0.929)	0.033
HER2 status			
Negative	1.0%	1	
Positive	1.2%	1.310 (0.539–3.184)	0.552
Chemotherapy			
Not done	0.6%	1	
Performed	1.2%	2.141 (0.728–6.294)	0.166

HR, hazard ratio; CI, confidence interval; BCS, breast-conserving surgery; HER2, human epidermal growth factor receptor 2

Supplementary Table S2-3. Effect of symptomatic VTE on the recurrence of breast cancer

	Univariable analysis		Multivariable analysis	
	HR (95% CI)	*p*	HR (95% CI)	*p*
Age group				
≤44	Reference			
45–59	0.827 (0.575–1.191)	0.308		
≥60	0.812 (0.515–1.282)	0.372		
T stage				
T_{is}	Reference		Reference	
1	6.016 (1.456–24.860)	0.013	4.651 (1.118–19.343)	0.035
2	22.697 (5.594–92.098)	<0.001	12.139 (2.936–50.196)	0.001
3	35.723 (7.827–163.046)	<0.001	15.454 (3.292–72.540)	0.001
4	67.903 (14.875–309.981)	<0.001	20.860 (4.408–98.715)	<0.001
N stage				
0	Reference		Reference	
1	2.393 (1.616–3.543)	<0.001	1.756 (1.170–2.634)	0.007
2	4.423 (2.766–7.075)	<0.001	2.476 (1.516–4.045)	<0.001
3	15.062 (9.831–23.074)	<0.001	8.817 (5.558–13.987)	<0.001
Hormonal receptor				
Negative	Reference		Reference	
Positive	0.380 (0.278–0.519)	<0.001	0.383 (0.275–0.533)	<0.001
HER2 status				
Negative	Reference		Reference	
Positive	1.229 (0.875–1.727)	0.234	0.658 (0.459–0.944)	0.023
Symptomatic VTE				
Absent	Reference		Reference	
Present	6.935 (3.405–14.128)	<0.001	3.120 (1.502–6.482)	<0.001

HR, hazard ratio; HER2, human epidermal growth factor receptor 2

Supplementary Table S2-4. Effect of symptomatic VTE on the mortality of the patients

	Univariable analysis		Multivariable analysis	
	HR (95% CI)	*p*	HR (95% CI)	*p*
Age group				
≤44	Reference			
45–59	1.213 (0.642–2.293)	0.552		
≥60	2.058 (1.041–4.071)	0.038		
T stage				
T_{is}	Reference			
1	1.637 (0.482–5.565)	0.430		
2	4.919 (1.510–16.029)	0.008		
3	11.336 (2.835–45.339)	0.001		
4	28.803 (7.796–106.418)	<0.001		
N stage				
0	Reference		Reference	
1	0.890 (0.364–2.179)	<0.799	0.935 (0.382–2.293)	0.884
2	5.787 (2.945–11.374)	<0.001	4.2232 (2.114–8.435)	<0.001
3	24.380 (14.098–42.161)	<0.001	13.822 (7.436–25.692)	<0.001
M stage				
0	Reference		Reference	
1	19.667 (11.159–34.661)	<0.001	4.923 (2.581–9.393)	<0.001
Hormonal receptor				
Negative	Reference		Reference	
Positive	0.293 (0.184–0.466)	<0.001	0.290 (0.179–0.468)	<0.001
HER2				
Negative	Reference			
Positive	1.780 (1.102–2.874)	0.018		
Symptomatic VTE				
Absent	Reference		Reference	
Present	11.327 (4.904–26.163)	<0.001	4.583 (1.946–10.791)	<0.001

HR, hazard ratio; HER2, human epidermal growth factor receptor 2
